# αTAT1-induced tubulin acetylation promotes ameloblastoma migration and invasion

**DOI:** 10.1038/s41374-021-00671-w

**Published:** 2021-09-10

**Authors:** Shohei Yoshimoto, Hiromitsu Morita, Kazuhiko Okamura, Akimitsu Hiraki, Shuichi Hashimoto

**Affiliations:** 1 Section of Pathology, Department of Morphological Biology, Division of Biomedical Sciences, Fukuoka Dental College, Fukuoka, 814-0193 Japan; 2 Oral Medicine Research Center, Fukuoka Dental College, Fukuoka, 814-0193 Japan; 3 The Center for Visiting Dental Service, Department of General Dentistry, Fukuoka Dental College, Fukuoka, 814-0193 Japan; 4 Section of Oral Oncology, Department of Oral and Maxillofacial Surgery, Division of Oral and Medical Management, Fukuoka Dental College, Fukuoka, 814-0193 Japan

**Keywords:** Oral cancer, Cell invasion

## Abstract

Ameloblastoma (AB) is the most common benign epithelial odontogenic tumor occurring in the jawbone. AB is a slowly growing tumor but sometimes shows a locally invasive and an aggressive growth pattern with a marked bone resorption. In addition, the local recurrence and distant metastasis of AB also sometimes occurs, which resembles one of the typical malignant potentials. From these points of view, to understand better the mechanisms of AB cell migration or invasion is necessary for the better clinical therapy and improvements of the patients’ quality of life. Microtubules in eukaryotic cells reveal the shape of hollow cylinders made up of polymerized alpha (α)- and beta (β)-tubulin dimers and form the cytoskeleton together with microfilaments and intermediate filaments. Microtubules play important roles in cell migration by undergoing assembly and disassembly with post-translational modifications. Stability of microtubules caused by their acetylation is involved in cell migration. In this study, we investigated the expression and distribution of acetylated α-tubulin and alpha-tubulin N-acetyltransferase 1 (αTAT1), an enzyme which acetylates Lys-40 in α-tubulin, in AB specimens, and analyzed how tubulin was acetylated by αTAT1 activation in a human AB cell line, AM-1. Finally, we clarified that TGF-β-activated kinase1 (TAK1) was phosphorylated by TGF-β stimulation, then, induced tubulin acetylation via αTAT1 activation, which subsequently activated the migration and invasion of AB cells.

## Abstract (web summary)

Ameloblastoma (AB) is the most common benign odontogenic tumor. In this study, we investigated the expression and distribution of acetylated α-tubulin and αTAT1 in AB specimens. We analyzed tubulin acetylation caused by αTAT1 activation in a human AB cell line, AM-1. Results of the histopathological and in vitro studies clarified that TAK1 was phosphorylated by TGF-β stimulation, then, induced tubulin acetylation via αTAT1 activation, which subsequently activated the migration and invasion of AB cells.

## Introduction

Ameloblastoma (AB) is the most common benign epithelial odontogenic tumor occurring in the mandibular or maxillary bones^1^. The growth pattern of AB is usually slow, but sometimes shows an aggressive appearance with a locally invasive growth and bone resorption^2^. Because of its invasive growth, surgical resection is generally selected as the first choice for the treatment. However, the local recurrence or distant metastasis of this tumor sometimes occurs, which resembles one of the typical malignant potentials. From these points of view, to understand better the mechanisms of AB cell invasion or migration could contribute to the development of the better therapeutic strategy and potential biomarkers for the diagnosis and finally to the improvement of the patients’ quality of life.

In cell migration, it is well known that cytoskeletons, such as actin filaments and microtubules, play important roles. Microtubules in eukaryotic cells reveal the shape of hollow cylinders made up of polymerized alpha (α)- and beta (β)-tubulin dimers and form the cytoskeleton together with microfilaments and intermediate filaments. Microtubules play important roles in cell migration by undergoing assembly and disassembly with post-translational modifications. Stability of microtubules caused by their acetylation is involved in cell migration^3–5^. Alpha-tubulin N-acetyltransferase 1 (αTAT1), an enzyme which acetylates Lys-40 in α tubulin, contributes microtubules stability^5^. In the metastatic breast cancer, it was reported that elevated levels of α-tubulin acetylation were correlated with poor prognosis^6^. In colon cancer, it was also reported that the increased expression of αTAT1 contributed to the cancer cell invasion^7^. However, in the head and neck tumors progression, roles of tubulin acetylation remain unclear.

In this study, we investigated the expression and distribution of acetylated α-tubulin and αTAT1 in AB specimens. Moreover, we analyzed tubulin acetylation caused by αTAT1 activation in a human AB cell line, AM-1. Results of the histopathological and in vitro studies clarified that TGF-β-activated kinase1 (TAK1) was phosphorylated by TGF-β stimulation, then, induced tubulin acetylation via αTAT1 activation, which subsequently activated the migration and invasion of AB cells.

## Materials and methods

### Patients and clinicopathological profiles

This clinical study using the patients’ information was done under the permission of the ethics committee in Fukuoka Dental College (ID number: 339). The patients had singed a written informed consent form or had been given an opportunity to opt-out. 32 cases of AB (Male/Female: 22/10, mean age: 35.8, range: 15–66) were examined. These Japanese patients underwent surgery at Fukuoka Dental College Hospital, Fukuoka, Japan, between 2010 and 2018. Neither chemotherapy nor irradiation was prescribed for the patients before surgery. Tissue samples were stored as formalin-fixed and paraffin-embedded specimens. The pathological diagnosis was done by at least two pathologists at the department of pathology, Fukuoka Dental College Hospital, Fukuoka, Japan. The histological classification was performed according to the criteria of the “2017 WHO Classification of Head and Neck Tumors”^1^. The clinicopathological profiles of the patients are summarized in Table 1.

**Table 1:** Summary of the clinicopathological characteristics of ameloblastoma patients examined.

Characteristics		Number of patients	Immunohistochemical score (range: 0–3)	
Age	Mean: 35.8 (range: 15–66)			
Sex	Male	22		
	Female	10		
Pathological diagnosis			Ac-Tubulin	αTAT1
Conventional type AB	Follicular	16	2.06	1.94
	Plexiform	8	2.25	1.25
Unicystic AB		8	2.25	1.75
Total		32	2.16	1.72

The 32 cases of ameloblastoma (Male/Female: 22/10, mean age: 35.8; range: 15–66) examined in this study. The pathological diagnosis reveals as follows: ameloblastoma (AB), conventional type: 24 (Follicular type: 16, Plexiform type: 8), Unicystic AB: 8. Immunohistochemical scores (range: 0–3, average) of acetylated α-tubulin (Ac-Tubulin) and αTAT1 are shown.

### Antibodies

The primary antibodies; mouse anti-Acetylated Tubulin antibody (Ac-Tubulin; T7451), rabbit anti-αTAT1 antibody (HPA050999) and mouse anti-β-Actin antibody (T5441) were purchased from Sigma-Aldrich (St.Louis, MO, USA). Rabbit anti-TAK1 antibody (#4505), rabbit anti-phosphor-TAK1 (Ser412) antibody (#9339), and rabbit anti-E-cadherin antibody (#3195) were purchased from Cell Signaling Technology Inc. (Danvers, MA, USA). Rabbit anti-alpha Tubulin antibody (#11224-1-AP) were purchased from Proteintech (Rosemont, IL, USA). Mouse anti-αTAT1 antibody were purchased from Antibodies Incorporated (Davis, CA, USA). The secondary antibodies; horseradish peroxidase (HRP)-conjugated polymer anti-rabbit and -mouse antibodies were purchased from DAKO-Agilent Technologies Co. (Santa Clara, CA, USA). HRP-linked anti-rabbit and -mouse antibodies were purchased from Cell Signaling Technology Inc. Alexa Fluor 594 Phalloidin, Alexa Fluor 594-conjugated goat anti-rabbit IgG and Alexa Fluor 488-conjugated goat anti-mouse IgG antibodies were purchased from Thermo Fisher Scientific (Waltham, MA, USA).

### Immunostaining for tissues and cells

10% neutral buffered formalin-fixed and paraffin-embedded tissue blocks were cut into 4 μm-thick sections for H.E. and immunohistochemical staining. Antigen retrieval was performed for all sections by an autoclave treatment at 121 °C for 5 min in 0.01 M citrate buffer, pH 6.0. Immunostaining was performed by using EnVision/HRP kit (DAKO). Briefly, the sections were treated with a 0.1% hydrogen peroxide-methanol solution to inhibit endogenous peroxidase activity and a 5% BSA/TBS to block any nonspecific binding of primary antibodies. Subsequently, each section was incubated with the primary antibody against Ac-Tubulin (1:200 dilution), αTAT1 (1:100 dilution) or E-cadherin (1:200 dilution) at 4 °C overnight. These sections were then incubated with HRP conjugated polymer anti-rabbit or anti-mouse antibody. The peroxidase activity was visualized using 0.1% 3, 3′-diaminobenzidine and 0.01% hydrogen peroxide in TBS. For the immunofluorescent staining, after incubation with each primary antibody, the section was incubated with Alexa Fluor 594-conjugated goat anti-rabbit IgG (1:1,000 dilution) or Alexa Fluor 488-conjugated goat anti-mouse IgG (1:1,000 dilution) secondary antibody. Then, sections were mounted using VECTASHIELD with DAPI (Vector Lab., Burlingame, CA, USA). Photomicrographs were visualized and captured at the appropriate wavelength using a fluorescence microscope (LSM 710, Carl Zeiss Inc.). The images were processed using a ZEN 2010B Sp1 Ver. 6.0.0.485 software (Carl Zeiss Inc.). For immunocytochemistry, the same immunostaining procedure described above was applied for cells after fixation with 4% paraformaldehyde. Intensity of fluorescence was quantified using ImageJ^8^.

### Immunohistochemical assessment

Staining intensity was described as immunohistochemical score (IS) (scored on a scale of 0–3; 0: negative, 1: weakly positive (WP), 2: intermediately positive (IP), 3: strongly positive (SP)). Then, we defined the expression ratio (ER) as the ratio of the case number showing the positivity of at least WP against the total case number of each group. For the correlation analysis between Ac-Tubulin and αTAT1 expression, IS was statistically compared.

### Cell culture

The human AB cell line AM-1 was established from a plexiform-type AB representing typical features of native cells^9^, and was kindly donated by Dr. Mitsuyasu (Kyushu University, Fukuoka, Japan). Cells were grown in defined keratinocyte serum-free medium (D-KSFM; Invitrogen, San Diego, CA, USA) or Dulbecco’s Modified Eagle’s Medium (DMEM; SIGMA-Aldrich), supplemented with 10% fetal bovine serum (FBS, PAA Laboratories, Pasching, Austria) and incubated at 37 °C, 5% CO_2_. In time-course analyses, cells were incubated in DMEM with 10 ng/ml transforming growth factor-beta 1 (TGF-β1; Sigma-Aldrich), 100 ng/ml tumor necrosis factor-α (TNF-α; Sigma-Aldrich) or 100 ng/ml lipopolysaccharide (LPS) from *Porphyromonas gingivalis* (Sigma-Aldrich). For the inhibition of TAK1 expression, cells were preincubated in DMEM with 100 nM NG25 (Selleck, Houston, TX) at 37 °C, 5% CO_2_ for 1 h.

### Western blotting analysis

Cells were homogenized in an ice-cold lysis buffer and centrifuged at 50,000 × *g* for 30 min at 4 °C. Total protein of 20 μg from the supernatant was applied in each lane and was separated on a 4–12% Bis-Tris Plus gel (Thermo Fisher Scientific), then was transferred to a polyvinyldifluoride membrane (Merck Millipore, Darmstadt, Germany). Immunoblot analyses were performed using anti-Ac-Tubulin, anti-αTAT1, anti-TAK1 and anti-phospho-TAK1 primary antibodies (1:1000). Mouse anti-β-actin antibody (1:5,000) and rabbit anti-alpha Tubulin antibody (1:2000) were used for each internal standard detection. After the incubation with the primary antibodies, transblots were developed with HRP-linked secondary antibodies (1:3,000) and visualized by the enhanced chemiluminescence (ECL) system using ImmunoStar Zeta (Wako, Osaka, Japan), and the bands were scanned and imaged by LAS-4000 (GE Healthcare, Little Chalfont, UK).

### RNA interference

Protein knockdown was carried out using small interfering RNA (siRNA) oligonucleotides, siATAT1 (siRNA for αTAT1) (#1: 5′-CAGAAUCUUUCCGCUCCUA-3′ and 5′-UAGGAGCGGAAAGAUUCUG-3′, #2: 5′-GAGUAACCGCCAUGUUGUU-3′ and 5′-AACAACAUGGCGGUUACU-3′, Sigma-Aldrich) or siTAK1(Duplex sequences, #A: rGrCrUrGrArCrArUrGrUrCrUrGrArArArUrArGrArArGrCTA, #B: rArArUrCrUrGrArGrArGrGrArArArGrCrGrUrUrUrArUrUGT, OriGene Technologies, Rockville, MD, USA). For negative control, Scramble siRNA (siNeg) was purchased from Sigma-Aldrich or OriGene. siRNA was transfected into AM-1 cells at 25 pmol/125 μl final concentrations with Screen Fect A plus (Wako), using a forward transfection method according to the manufacture’s protocols.

### Wound healing assay

Wounds were prepared by using Culture-Insert (two well; ibidi, Madison, WS, USA). AM-1 cells were cultured for 24 h in an objective condition, then the Culture-Insert was removed. After the consecutive 22 h sustained culture, the area of remaining wounds was determined using Image J (National Institutes of Health, Bethesda, MD, USA). The closing ratio (CR) of the wounded area was calculated by the following formula; CR = (w−rw)/w × 100 (%) (w: wounded area at the start point, rw: remaining wounded area). Then, the mean value of the CR in each condition was statistically compared.

### Cell migration and invasion assay

AM-1 cells which were cultured in serum free DMEM with siNeg or siATAT1 were seeded into each insert of the 24-well Falcon culture insert (#353097, Corning, NY, USA) for the migration assay, or seeded into each insert of the 24-well Biocoat Matrigel Invasion Chamber (#351180, Corning) for the invasion assay. FBS was added at the final concentration of 10% in each 24-well plate outer chamber to induce cell migration or invasion. After the 24 h incubation, the cells remaining on the surface of the insert membranes were carefully removed with cotton swabs, and the cells that had migrated or invaded to the opposite sides of the membranes were stained with the Diff-Quik Kit (Sysmex Corp., Kobe, Japan). After staining, cells were counted using BZ-X710 microscopy (KEYENCE, Tokyo, Japan).

### 3D spheroid culture

1 × 10^4^ cells in 200 μl DMEM with 0.01 mg gelatin powders (Genocel Powder type, NikkeMedical, Osaka, Japan) were seeded in each well of 96-well Nunclon Sphera plate (Thermo Fisher Scientific). The spheroid formation processes were observed at least every 24 h to monitor morphological changes.

### Statistical analysis

All data were expressed as the mean ± standard error of the mean (SEM). Unpaired Student’s *t* tests and Mann–Whitney U tests were applied for the comparison between two groups. For the correlation analysis, Kolmogorov–Smirnov normality test and Pearson correlation coefficients were used. Statistical significance was set as *p < 0.05, **p < 0.01 and ***p < 0.001.

## Results

### Immunohistochemical expression of acetylated α-tubulin and αTAT1 in ameloblastoma

**Fig. 1:**
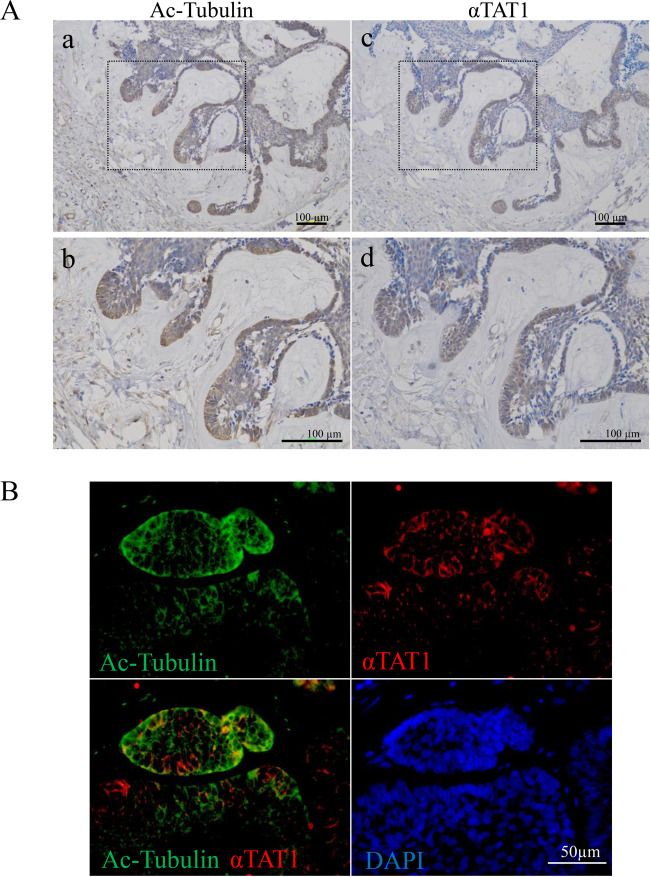
Immunohistochemical analyses of acetylated α-tubulin and αTAT1 expressions in ameloblastoma. **A** Acetylated α-tubulin (Ac-Tubulin; a, b) and αTAT1 (c, d) expressions are shown. Magnified images are shown in the lower panels (b, d). Both Ac-tubulin and αTAT1 expressions were seen in tumor cells especially at the tip of the budding region and invasive front. Scale bars: 100 μm. **B** Dual immunohistochemical staining of Ac-tubulin (green) and αTAT1 (red) in ameloblastoma. Co-expression of Ac-tubulin and αTAT1 is apparent in the cytoplasm of tumor cells especially at the tip of the invasive front. Nuclei are stained with DAPI (blue). Scale bars: 50 μm.

We first examined protein expression and distribution of acetylated α-tubulin and αTAT1 in 32 AB cases (Table 1). Immunohistochemically, both acetylated α-tubulin and αTAT1 were observed almost all specimens [acetylated α-tubulin; ER = 100% (32/32 cases), IS = 2.16, αTAT1: ER = 87.5% (28/32 cases), IS = 1.72]. There were no significant differences in the ISs between three types of AB, follicular, plexiform and unicystic AB. Moreover, a positive correlation was found between acetylated α-tubulin and αTAT1 in the IS of each specimen (Pearson correlation coefficients: *r* = 0.558, *p* < 0.01) (Table 1). We also examined the expressions of acetylated α-tubulin and αTAT1 in nine cases of dentigerous cyst as a control. Immunohistochemically, the ER and IS mean were both lower than those of AB, respectively [acetylated α-tubulin; ER = 44.4% (4/9 cases), IS = 0.44 (*p* < 0.0001), αTAT1; ER = 22.2% (2/9 cases), IS = 0.22 (*p* < 0.0001)] (Supplementary table 1, Supplementary Fig. 1A). In the distribution analyses, both acetylated α-tubulin and αTAT1 expressions were observed in the tumor cells mainly at the tip of the budding region and the invasive front (Fig. 1A, Supplementary Fig. 1B). Furthermore, co-expression of acetylated α-tubulin and αTAT1 in the cytoplasm of tumor cells was apparent especially at the tip of the invasive front (Fig. 1B). These findings suggested that tubulin acetylation was closely related to the αTAT1 expression and played a key role in the AB cell invasion and tumor progression.

### Tubulin acetylation induced by αTAT1 contributed to the cell migration and invasion in AM-1 cells

**Fig. 2:**
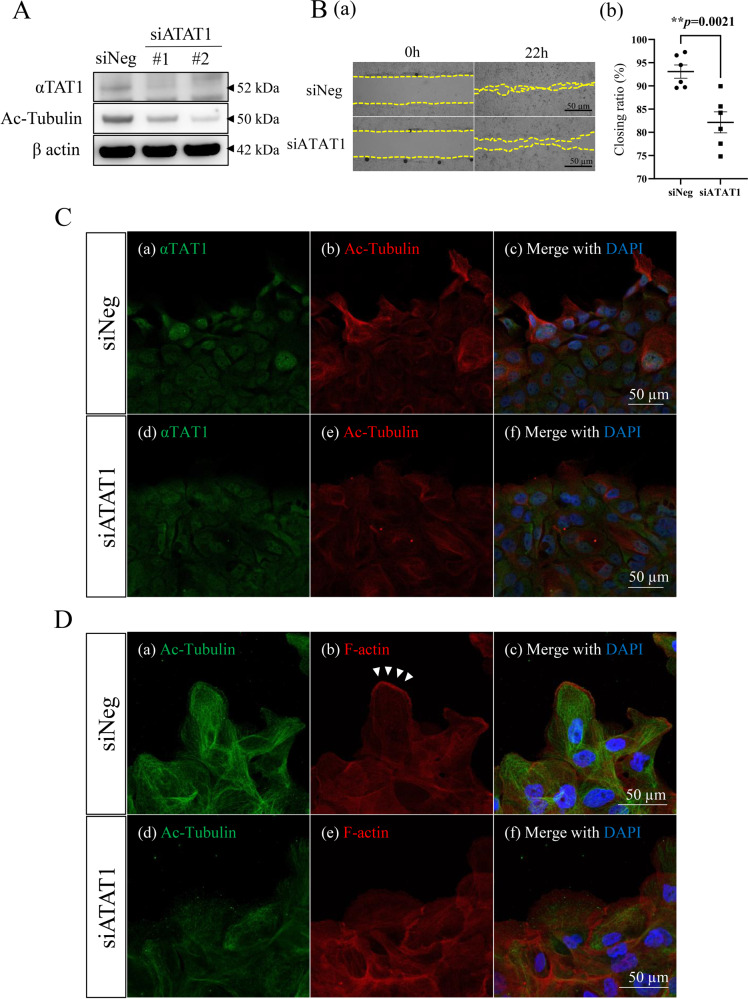
Analyses of αTAT1-induced tubulin acetylation on the cell migration in AM-1 cells. **A** Western blots of αTAT1 and Acetylated α-tubulin (Ac-Tubulin) in control (scramble siRNA transfected; siNeg) and αTAT1 knocked down cells (siATAT1). **B** (a) Wound healing assay to evaluate the migration ability of AM-1 cells in the control (siNeg) and siATAT1 transfected conditions. The front line of migrative AM-1 cells is represented by a yellow dotted line in each panel. The culture is stopped after 22 h. Scale bars: 50 μm. b Each dot in the graph indicates the closing ratio of the wounded area in siNeg or siATAT1 transfected condition. Statistical significance was set as **p < 0.01 (*n* = 6). **C** Dual immunocytostaining of αTAT1 (green: a, d) and Ac-Tubulin (red: b, e) in the control (siNeg) (a–c) and siATAT1 transfected (d–e) cells. Merged images with DAPI staining are shown in (c, f). Scale bars: 50 μm. **D** Dual immunocytostaining of Ac-Tubulin (green: a, d) and F-actin (red: b, e) in the control (siNeg) (a–c) and siATAT1 transfected (d–f) cells. Merged images with DAPI staining are shown (c, f). Allow heads show lamellipodium formation at the leading edge of the migrating cells. Scale bars: 50 μm.

**Fig. 3:**
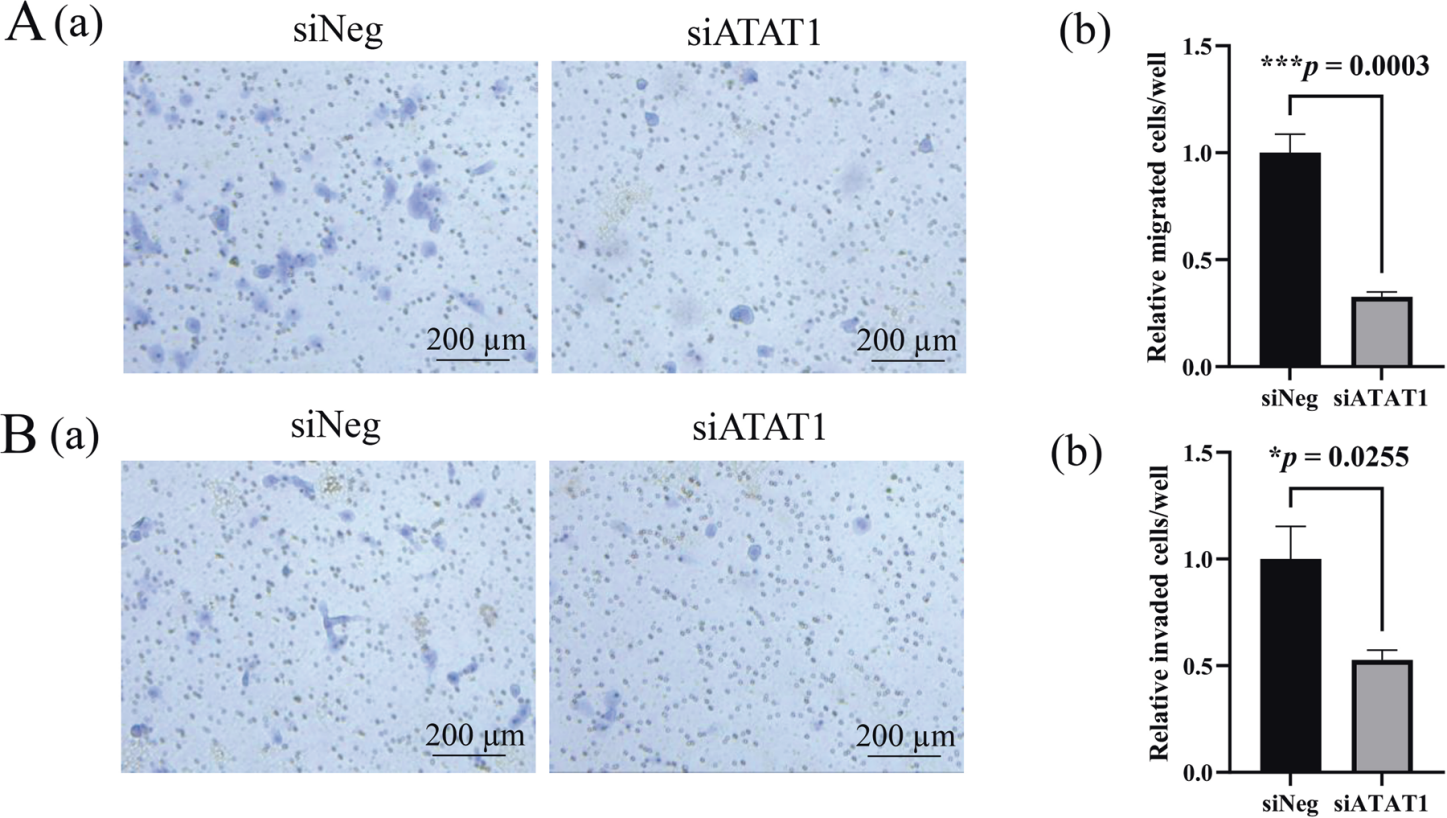
Functional analyses of tubulin acetylation on the cell migration and invasion in AM-1 cells by the inhibition of αTAT1. **A** (a) Transwell cell migration assay for control (siNeg) and siATAT1 transfected cells. Scale bars: 200 μm. b Bars indicate relative migrated cells in control (siNeg) and siATAT1 transfected cells. Statistical significance was set as ***p < 0.001 (*n* = 4). **B** (a) Transwell cell invasion assay for the control (siNeg) and siATAT1 transfected cells. Scale bars: 200 μm. b Bars indicate relative invaded cells in the control (siNeg) and siATAT1 transfected cells. Statistical significance was set as *p < 0.05 (*n* = 4).

To clarify the role of tubulin acetylation in AB, we performed in vitro tumor cell migration and invasion analyses using a human AB cell line, AM-1. First, we suppressed αTAT1 protein expression using siRNA (siATAT1) (*p* = 0.0010, *p* = 0.000066) (Supplementary Fig. 2). Western blotting analyses showed that α-tubulin acetylation was suppressed in αTAT1 knocked down cells (Fig. 2A). Based on the result that the co-expression of acetylated α-tubulin and αTAT1 immunoreaction was seen in the tumor cells especially at the tip of the invasive front of AB (Fig. 1A and B), we hypothesized that αTAT1 and tubulin acetylation might be significantly related to the tumor cell migration or invasion in AB. In the wound healing assays, cell migration ability, namely, the closing ratio of wounded area (CR), was significantly decreased by siATAT1 transfection (Fig. 2B; a, b; *p* = 0.0021). Immunofluorescent cytochemical staining also revealed that acetylated α-tubulin and αTAT1 were dual positive in the migrating cells at the closing front of the wounded area in control cells (scramble siRNA transfected; siNeg) (Fig. 2C; a–c). On the other hand, siATAT1 transfected cells showed decrease of acetylated α-tubulin staining (Fig. 2C; d–f). Moreover, migrating siNeg-transfected cells showed positive staining of acetylated α-tubulin and F-actin staining at the leading edge, suggesting lamellopodium formation (Fig. 2D; a–c). In siATAT1-transfected cells, both acetylated α-tubulin and F-actin expressions were downregulated (Fig. 2D; d–f). The transwell cell migration and invasion assays also showed that αTAT1 knockdown significantly repressed the mobility of AM-1 cells (Fig. 3A; a, b; *p* = 0.0003, Fig. 3B; a, b; *p* = 0.0255). These results indicated that tubulin acetylation was induced by αTAT1, then contributed to the AM-1 cell migration and invasion.

### TAK1 activation triggered tubulin acetylation in AM-1 cells

**Fig. 4:**
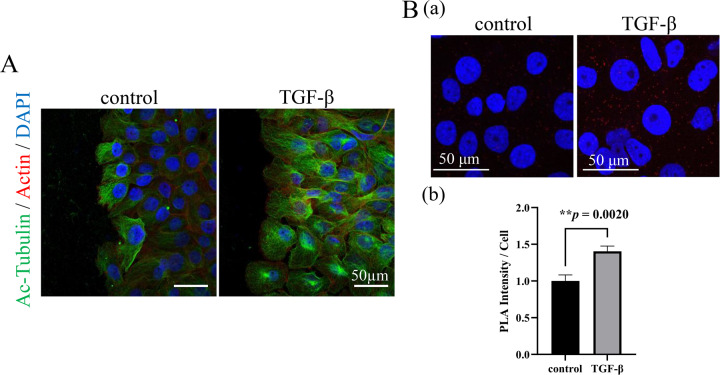
Analyses of the effect of TGF-β stimulation and the mechanisms of related molecules interaction on the tubulin acetylation in AM-1 cells. **A** Immunocytostaining of Ac-Tubulin (green) and F-actin (red) in the control and TGF-β stimulated AM-1 cells. Nuclei are stained with DAPI (blue). Ac-Tubulin expression is upregulated by the TGF-β stimulation. Scale bars: 50 μm. **B** (a) In situ proximity ligation assay to confirm the interaction of αTAT1 and TAK1. Colocalization of αTAT1 and TAK1 which are secondary labeled by PLA probes (red dots) in the control and TGF-β stimulated conditions. Nuclei are stained with DAPI (blue). Scale bars: 50 μm. b Bars indicate relative PLA intensity in the control and TGF-β stimulated conditions. Statistical significance was set as **p < 0.01 (*n* = 9).

**Fig. 5:**
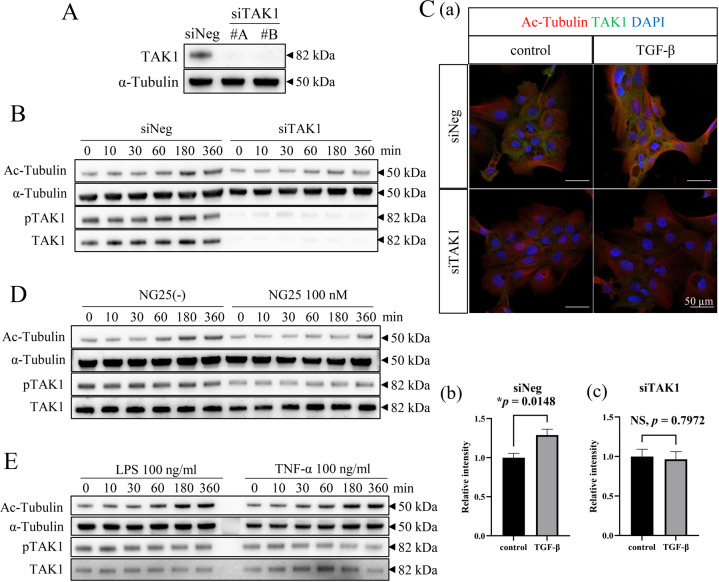
Analyses of the effect of activated/phosphorylated-TAK1 on the tubulin acetylation in AM-1 cells by the inhibition of TAK1. **A** Western blots of TAK1 and α-tubulin in control (scramble siRNA transfected; siNeg) and TAK1 knocked down cells (siTAK1). **B** Time course analysis of α-tubulin acetylation and TAK1 phosphorylation by the TGF-β stimulation in the control (siNeg) and TAK1 knocked down cells (siTAK1). In TAK1 knocked down cells, TGF-β-induced tubulin acetylation and TAK1 phosphorylation are suppressed. **C** (a) Immunocytostaining of Ac-Tubulin (red) and TAK1 (green) in the control (siNeg) and TAK1 knocked down cells (siTAK1) with or without TGF-β stimulation. Nuclei are stained with DAPI (blue). Scale bars: 50 μm. Bars indicate relative intensity of Ac-Tubulin in control (siNeg; b) and TAK1 knocked down cells (siTAK1; c). TGF-β-induced tubulin acetylation is significantly suppressed in TAK1 knocked down cells. Statistical significance was set as *p < 0.05 (*n* = 5). **D** Time course analysis of α-tubulin acetylation and TAK1 phosphorylation by the TGF-β stimulation in the control or NG25, an TAK1 inhibitor, treated cells. The similar result as seen in (**B**) is acquired. **E** Time course analysis of α-tubulin acetylation and TAK1 phosphorylation by the LPS (100 ng/ml) or TNF-α (100 ng/ml) stimulation in AM-1 cells. TAK1 phosphorylation and tubulin acetylation in AM-1 cells are also upregulated by the lipopolysaccharide (LPS) and tumor necrosis factor-α (TNF-α) stimulations.

Tumor development generally depends on the surrounding environment, known as tumor microenvironment (TME). In the TME, transforming growth factor-β (TGF-β) is secreted by stromal fibroblasts, macrophages, endothelial cells, and tumor cells^10–12^. Based on our previous report that TGF-β was expressed in AB cells under the hypoxic condition in the TME^13^, we next investigated how TGF-β stimulation affected the tubulin acetylation in AM-1 cells. Immunofluorescent cytostaining analyses revealed that tubulin acetylation was remarkably upregulated by the TGF-β stimulatation in AM-1 cells (Fig. 4A). One previous study demonstrated that TAK1 was a pivotal kinase in TGF-β induced tubulin acetylation by αTAT1^14^. From these points of view, we next investigated the interaction between TAK1 and αTAT1 in AM-1 cells. We performed proximity ligation assay to assess whether TAK1 was bound to αTAT1, and found that TGF-β stimulation promoted colocalization of TAK1 and αTAT1 (Fig. 4B; *p* = 0.0020). To determine the effect of TAK1 in tubulin acetylation, we suppressed TAK1 protein expression using siRNA (Fig. 5A). In scramble siRNA transfected control cells, TGF-β-induced upregulation of tubulin acetylation and TAK1 phosphorylation was observed in a time-dependent manner (Fig. 5B; siNeg). On the other hand, in TAK1 knocked down cells, TGF-β-induced tubulin acetylation and TAK1 phosphorylation were suppressed (Fig. 5B; siTAK1). Immunocytochemical analyses also indicated that TGF-β-induced tubulin acetylation was significantly suppressed in TAK1 knocked down cells (Fig. 5C; a, b; *p* = 0.0148, c; *p* = 0.7972). In addition, TAK1 phosphorylation and tubulin acetylation were also both inhibited by NG25, a TAK1 inhibitor (Fig. 5D). TAK1 activation has been reported to be caused by the signaling from some different cell surface receptors^15,16^. In this study, LPS and TNF-α stimulations also upregulated TAK1 phosphorylation and subsequent tubulin acetylation (Fig. 5E). These results indicated that TAK1 activation/phosphorylation triggered α-tubulin acetylation in AM-1 cells.

### TGF-β stimulation caused tubulin acetylation in a 3D culture model of AM-1 cells

**Fig. 6:**
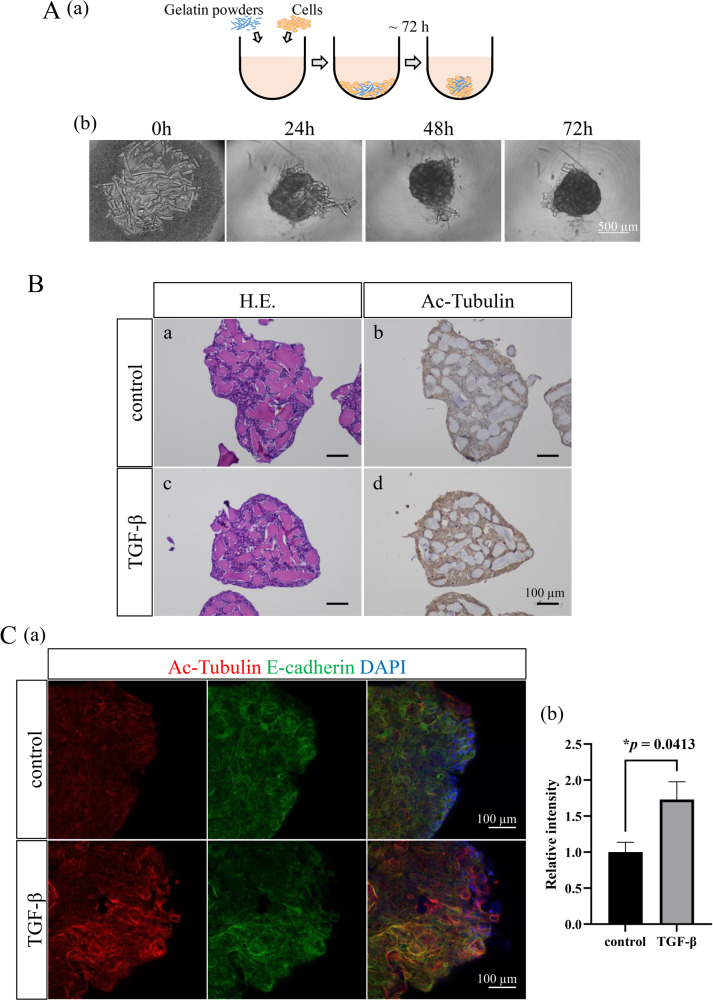
Analyses of the tubulin acetylation by the TGF-β stimulation in AM-1 cells using a 3D culture model. **A** (a) A scheme of the method to make AM-1 spheroids containing gelatin powders. b Time course analysis of a spheroid formation. Aggregation of cells and gelatin powders are seen within 24 h, and spheroids are formed within 72 h. Scale bars: 500 μm. **B** Sections of control (a, b) and TGF-β stimulated (c, d) spheroids. Spheroids are showing plexiform formation of AM-1 cells in Hematoxylin-Eosin (H.E.) staining (a, c). Immunohistostaining of Acetylated α-tubulin (Ac-Tubulin) in the control (b) and TGF-β stimulated (d) conditions. The expression of acetylated α-tubulin in AM-1 spheroids is upregulated by the TGF-β stimulation. Scale bars: 100 μm. **C** (a) Immunofluorescence staining of Ac-Tubulin (red) and E-cadherin (green) in the control and TGF-β stimulated spheroids. Nuclei are stained with DAPI (blue). Scale bars: 100 μm. b Bars indicate relative intensity of Ac-Tubulin in the control and TGF-β stimulated spheroids. The expression of acetylated tubulin is significantly increased in the spheroids especially at the outer layers by the TGF-β stimulation. Statistical significance was set as *p < 0.05 (*n* = 4).

It is well known that the in vivo tissue or tumor condition can be much better reflected in the three-dimensional (3D) culture models than in the conventional two-dimensional (2D) culture models. By this reason, we chose a 3D culture system to analyze the expression and distribution of proteins of interest in the tumor spheroids. To mimic the in vivo tissue growth and proliferation of AB, we established AM-1 spheres in the 3D culture system containing gelatin powders (Fig. 6A; a). When we cultured AM-1 cells with gelatin powders in U-bottom culture plates, aggregates of cells and gelatin powders were seen within 24 h, and compact spheres were formed within 72 h (Fig. 6A; b). By the hematoxylin-eosin (H.E.) staining, the spheroids consisted of AM-1 cells proliferating in plexiform pattern with aggregates of gelatin powders like tumor stroma, mimicking the in vivo AB tissues (Fig. 6B; a, c). TGF-β stimulation upregulated the expression of acetylated α-tubulin in AM-1 spheroids (Fig. 6B; b, d). Immunofluorescent staining also revealed a significant increase of the acetylated tubulin expression in the spheroids especially at the outer layers by the TGF-β stimulation (Fig. 6C; a, b; *p* = 0.0413). These results suggested that in the TMEs TGF-β produced by the stromal cells promoted tubulin acetylation of AB cells contributing to the AB progression.

## Discussion

**Fig. 7:**
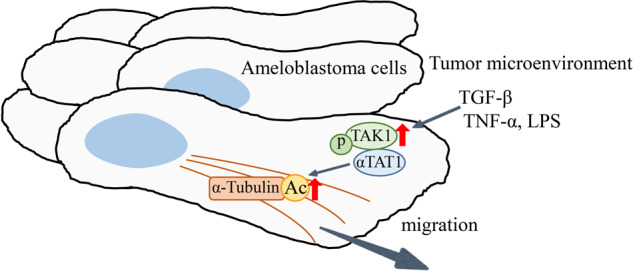
The schematic illustration of α-tubulin acetylation in AB cells. TGF-β, TNF-α, and LPS, inflammatory factors in the tumor microenvironment, stimulate AB cells by binding the cell surface specific ligands, then, the intra-cellular signaling activates TAK1 by inducing the phosphorylation. αTAT1 is activated by interacting with p-TAK1 and causes tubulin acetylation, which finally contributes to the migration and invasion of AB cells.

In this study, we clarified one of the mechanisms of the migration and invasion of AB cells in the tumor progression. The histopathological and immunohistochemical analyses for AB tissues and in vitro studies using AM-1 cells revealed that TGF-β stimulation activated TAK1 by inducing its phosphorylation, then, αTAT1 was activated by interacting with p-TAK1 causing tubulin acetylation, which finally contributed to the migration and invasion of AB cells (Fig. 7).

AB shows unique histopathological features similar to an enamel organ and a dental lamina in the developmental stages of tooth germ. So-called “reverse polarity” in peripheral cells of tumor nests is a typical histopathological feature of AB^1,17^. In our study, immunohistochemical staining revealed that both acetylated α-tubulin and αTAT1 expressions were observed in tumor cells showing reverse polarity in invasive fronts (Fig. 1A). In general, unicystic AB is known to be less invasive than conventional type AB. However, there were no significant differences in the IS of acetylated α-tubulin and αTAT1 between conventional type AB and unicystic AB (Table 1). Unicystic AB were classified as luminal, intraluminal, and mural subtypes^18^. It was reported that the recurrence rate of mural subtype was higher than that of other subtypes^18^. In our specimens, both acetylated α-tubulin and αTAT1 expressions were observed in the tumor cells mainly at the tip of the intramural growth of the cystic lesion and mural nests (Supplementary Fig. 1B). Thus, the results of our immunohistochemical staining were considered to suggest tumor cells dynamics in the local area. Wound healing assays by which collective cell migration ability could be analyzed also showed the expression of acetylated α-tubulin in the migrating cells with reverse polarity (Fig. 2C). These findings suggested that α-tubulin acetylation contributed migration of AB, and could be a potential biomarker of migration and/or invasion ability of AB. It has been reported that alterations in the acetylation levels of α-tubulin are controlled by αTAT1, acetyltransferase, and histone deacetylase 6 (HDAC6), deacetylase^19^. Some previous studies showed that HDAC6-mediated tubulin deacetylation also promoted microtube stabilization and cancer progression^19,20^. Therefore, microtube stabilization caused by a balance of tubulin acetylation might play a pivotal role in the progression of tumors including AB.

In a previous study using lung cancer cells, it was reported that tubulin acetylation upregulated the level of an antiapoptotic protein and contributed drug resistance^21^. From our in vitro experiments focusing on the relationship between tubulin acetylation and cell migration in AM-1 cells, we got a result corresponding to the pervious one that tubulin acetylation participated in anti-apoptosis and progression of AB. However, we need further studies to conclude that tubulin acetylation could be one of the therapeutic targets significantly effective for the tumor progression.

TAK1, a member of the mitogen-activated protein kinase kinase kinase family, was identified as an effector of TGF-β-induced p38/JNK pathway activation^22^. TAK1 plays some important roles in cellular homeostasis in various organs^23,24^. The activation of αTAT1 is induced by TAK1 phosphorylation due to some extracellular stimulation. TAK1 is not only activated/phosphorylated by TGF-β but also by LPS and TNF-α^15,16^. It is common knowledge that oral cavity is consistently inflamed associating with numerous bacteria. In our in vitro study using AM-1 cells, western blotting analyses revealed that LPS and TNF-α stimulations also upregulated TAK1 phosphorylation and subsequent tubulin acetylation (Fig. 5E). In the TME of oral squamous cell carcinoma, LPS from bacteria and inflammatory cytokines promote tumor progression^25,26^. Thus, control of inflammation in oral cavity also might be effective in the suppression of AB progression.

In our in vitro assays using AM-1 cells, both migration and invasion abilities were similarly and significantly inhibited by the αTAT1 knockout condition (Fig. 2D, E). Oh et al. reported that αTAT1 knockout suppressed α-tubulin acetylation and downregulated β-catenin-induced Matrix metalloproteinase-2 (MMP2) and MMP9 expressions in a colon cancer cell line^7^. Matrix metalloproteinases (MMPs) are known as critical factors in bone invasion of AB and contribute to the progression^27–29^. From these previous reports, αTAT1 might also play a role in the MMPs regulation of AB. Thus, further investigation is needed to confirm whether αTAT1 contributes MMPs modification in AB.

Establishment of 3D cell culture models has enabled to do various physiologic and pathological studies resembling in vivo condition. Spheroid culture is one of the 3D cell culture methods and is easy to be handled. We modified this method and made AM-1 spheres containing gelatin powders as stromal components (Fig. 6A; a). Immunostaining revealed that TGF-β stimulation upregulated the expression of acetylated α-tubulin in AM-1 spheroids (Fig. 5B, C). By our 3D culture method, AM-1 spheroids were constructed being intermingled with gelatin powders as stromal components and the 3D structures mimicked the in vivo structures of AB tissues without any central necrotic changes which were sometimes recognized in the spheroids constructed by the conventional 3D methods. Moreover, our 3D culture method was easy to be handled and was considered useful for the pathophysiological and immunohistochemical analyses. Thus, the spheroid models produced by our 3D culture method well mimicked the in vivo conditions and could be a useful tool for applying it to some xenograft disease models and drug screening systems. However, further in vivo experiments or in vitro experiments using some different AB cell lines other than AM-1, if applicable, would be needed for the better understanding of the AB pathophysiology.

In summary, we clarified one of the mechanisms of the migration and invasion of AB cells in the tumor progression revealing that TGF-β stimulation activated TAK1 by inducing its phosphorylation, then, αTAT1 was activated by interacting with p-TAK1 causing tubulin acetylation, which finally contributed to the migration and invasion of AB cells. Then, αTAT1-induced tubulin acetylation could be a potential marker for evaluating the migrative and invasive activity and become a new and an effective therapeutic target in ABs.

## Supplementary information

Supplementary figures and table
