# Supplementary material for: αTAT1-induced tubulin acetylation promotes ameloblastoma migration and invasion
Source: Lab Invest. 2021 Sep 10;102(1):80–9. doi: 10.1038/s41374-021-00671-w (PMC8695380; doi:10.1038/s41374-021-00671-w)
Supplement: Supplementary file 1 — Supplementary figures and table [file 41374_2021_671_MOESM1_ESM.pdf]

Supplementary Information

Supplementary materials and methods

This clinical study using the patients' information was done under the permission of the ethics committee in Fukuoka Dental College (ID number: 339). The patients had singed a written informed consent form or were given an opportunity to opt-out. 9 cases of dentigerous cyst (Male/Female: 5/4, mean age: 44.6, range: 129-67) were examined.

Supplementary figures and table

Supplementary Table 1: Summary of the clinicopathological characteristics of dentigerous cyst patients examined

Characteristics		Number of patients	Immunohistochemical score (range: 0-3)	
Age	Mean: 44.6 (range: 29-67)			
Sex	Male	5		
	Female	4		
Pathological diagnosis			Ac-Tubulin	αTAT1
Dentigerous cyst		9	0.44	0.22

Supplementary Table 1: **Summary of the clinicopathological characteristics of dentigerous cyst patients examined.** The 9 cases of dentigerous cyst (Male/Female: 5/4, mean age: 44.6, range: 129-67) were examined.

Supplementary Figure 1

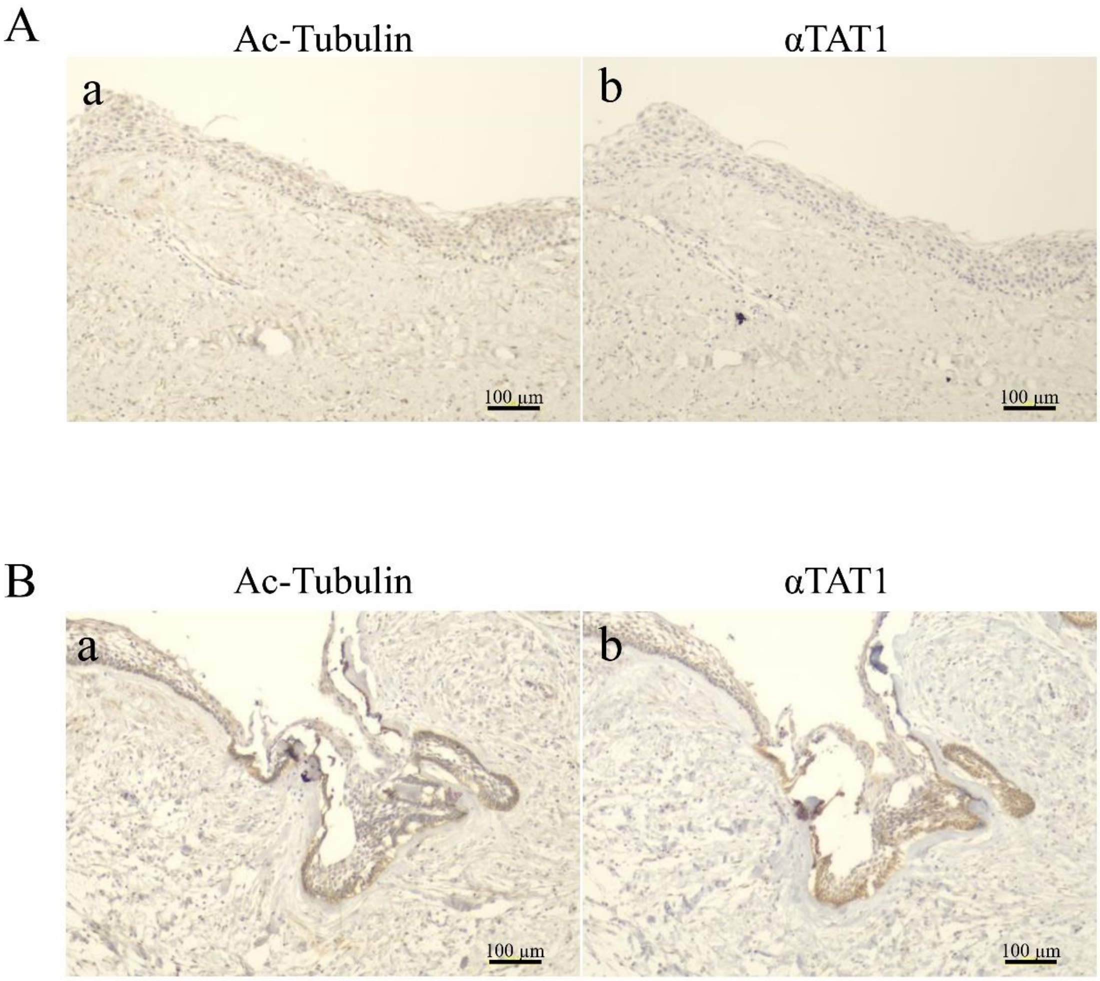

Supplementary Figure 1. **Immunohistochemical analyses of acetylated α-tubulin and αTAT1 expressions in dentigerous cyst and unicystic ameloblastoma.** A, Acetylated α-tubulin (Ac-Tubulin) (a) and αTAT1 (b) expressions in dentigerous cyst are shown. B, Acetylated α-tubulin (Ac-Tubulin) (a) and αTAT1 (b) expressions in unicystic ameloblastoma are shown. Scale bars: 100 µm.

Supplementary Figure 2

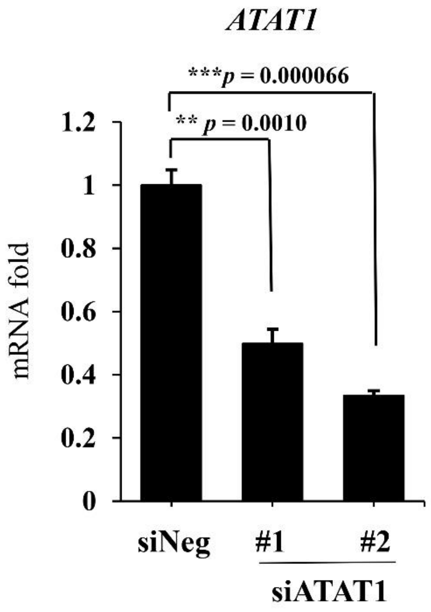

Supplementary Figure 2. **Inhibition analysis of ATAT1 mRNA expression with two different siRNAs specific for ATAT1 (siATAT1) on AM-1 cells.** Both #1 and #2 siRNAs statistically repress ATAT1 mRNA expression compared to that in control (siNeg). Statistical significance was set as *p<0.05 and ***p<0.001 (n=4).

Supplementary Figure 3

Figure 2A

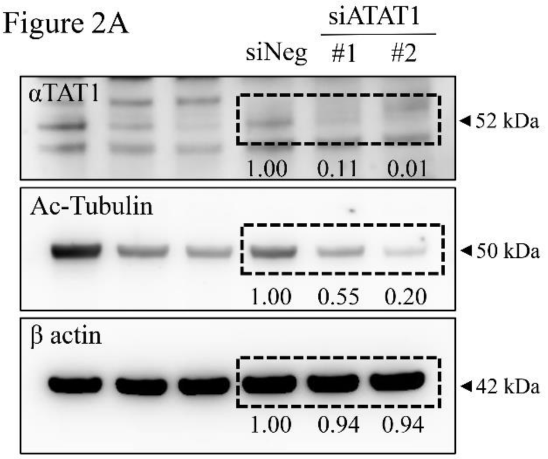

Figure 5A

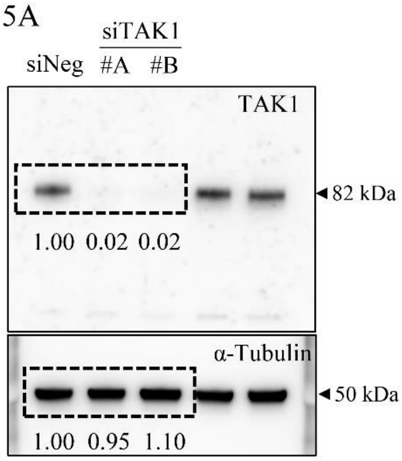

Figure 5B

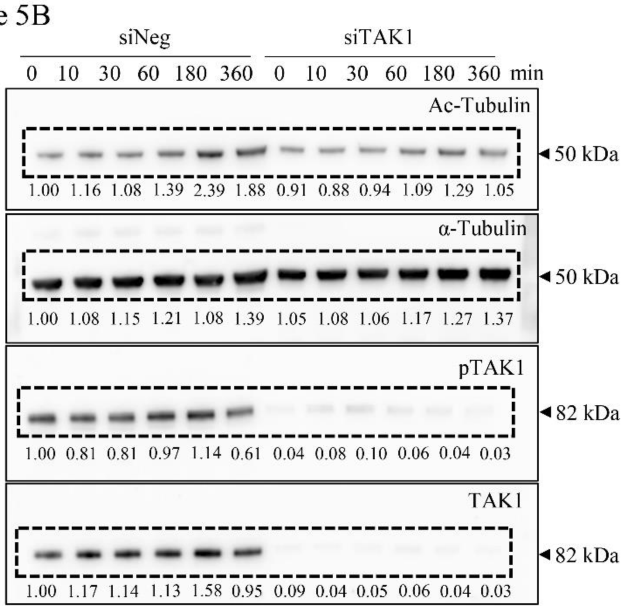

Supplementary Figure 3. **Original images of western blot.** Original images of western blot in Figure 2A, 5A and 5B. Images using in each figure are encircled by dotted lines. Intensity of blotting images are quantified using ImageJ, and each value is indicated under each band.

Supplementary Figure 4

Figure 5D

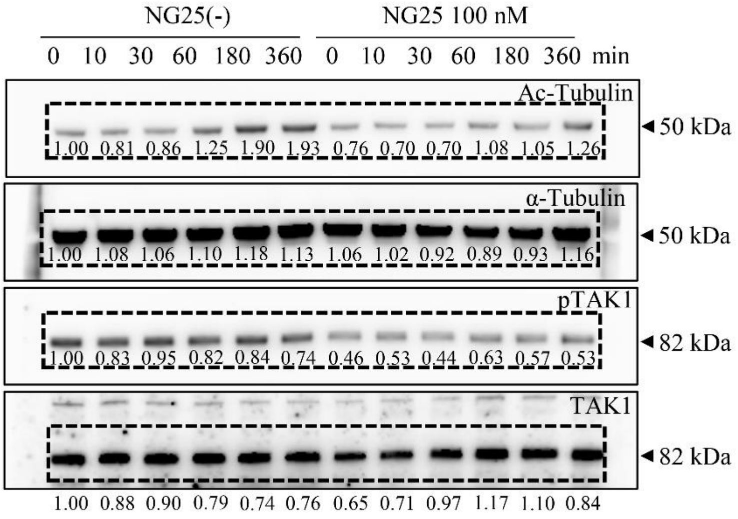

Figure 5E

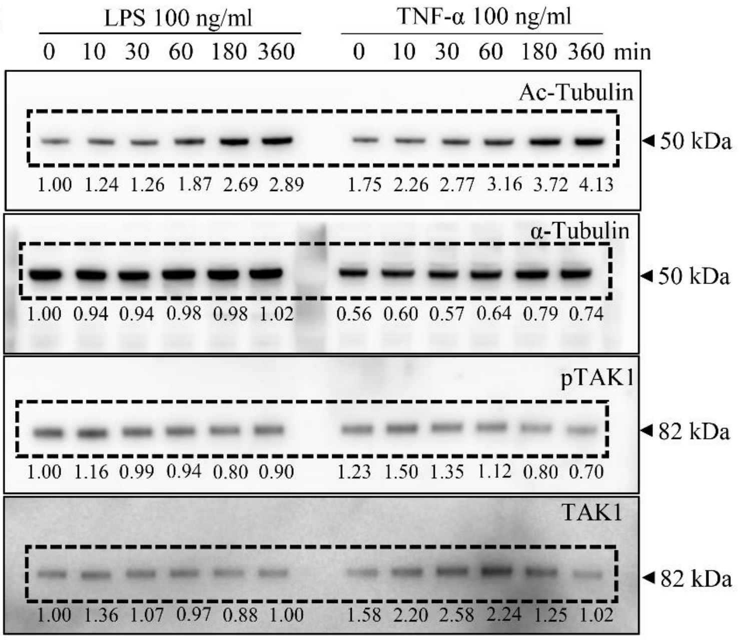

Supplementary Figure 4. **Original images of western blot.** Original images of western blot in Figure 5D and 5E. Images using in each figure are encircled by dotted lines. Intensity of blotting images are quantified using ImageJ, and each value is indicated under each band.
